# Editorial: Virus-induced innate immune response and inflammation

**DOI:** 10.3389/fmicb.2023.1213270

**Published:** 2023-06-06

**Authors:** Safder S. Ganaie, Zekun Wang, Bin Su, Sheema Mir

**Affiliations:** ^1^Department of Pathology and Immunology, Washington University in St. Louis, St. Louis, MO, United States; ^2^Joint National Laboratory for Antibody Drug Engineering, School of Basic Medical Sciences, Henan University, Kaifeng, China; ^3^Beijing Youan Hospital, Capital Medical University, Beijing, China; ^4^Western University of Health Sciences, Pomona, CA, United States

**Keywords:** inflammation, Early Growth Response 1, virus-induced innate immune response, type-I interferon response, Toll-like receptor-4

Host innate immune response acts as a first line of defense to counter the viral and/or pathogen invasion and subsequently activates the specific adaptive immune response against the invading pathogen (Dempsey et al., 2003; Chaplin, 2010). Innate immune response unlike adaptive immunity is not pathogen-specific, however, it recognizes conserved features of pathogens, known as pathogen-associated molecular patterns (PAMPs) (Mogensen, 2009; Kumar et al., 2011). The recognition of PAMPs requires sensors known as pattern-recognition receptors (PRRs), expressed by the cells of innate immunity and several other epithelial cells. PRRs include retinoic acid-inducible gene –I (RIG-I) like receptors (RLRs), C-type lectin-like receptors, Toll-like receptors (TLRs), and NOD-like receptors (NLRs) (Li and Wu, 2021). Once the PRRs recognize PAMPs, they are activated and results in cytokine and chemokine production, phagocytosis as well as antigen presentation to the adaptive immune system (Mogensen, 2009). In case of viral pathogens, PRRs recognize specific viral components such as viral RNA or DNA or viral intermediate products and induce type I interferons (IFNs) and other pro-inflammatory cytokines in the infected cells and other immune cells. However, over-activation of the innate immune response can cause systemic inflammation and tissue damage. Therefore, the controlled activation of innate immunity can be effective in thwarting the viral invasion without ensuing any host tissue damage. Though, viruses also have developed strategies to counter the antiviral state posed by the innate immune response by shutting down the PRR-induced pathways (García-Sastre, 2017; Ke and Yoo, 2017). Once the pathogen infection is established, it leads to the excessive production of proinflammatory cytokines that results in aggressive pro-inflammatory responses and insufficient control of anti-inflammatory responses that leads to a “cytokine storm,” which is one of the reasons for the increased mortality during virus infections (Fajgenbaum and June, 2020; Montazersaheb et al., 2022). In this issue, we invited articles that further our understanding of innate immune response against viral pathogens and the consequences thereof. More importantly, how viruses induce cytokine storms and cause severe inflammatory diseases like acute respiratory distress syndrome (ARDS) (Luyt et al., 2011). Under this Research Topic, we have a collection of seven manuscripts which include both research and review articles. These articles have highlighted the role of TLR4, EGR1, IFIT5, TLR2, IFNAR2, and viral proteins such as SARS-CoV2 NSP8 and HIV- capsid in innate immune response induced by viral pathogens.

The review article by Halajian et al. summarized the role of viral glycoproteins in the Toll-like receptor 4 (TLR4) activation. TLR4 recognizes multiple viral ligands and activates downstream signaling pathways and induces cytokine storm. The authors have nicely summarized how TLR signaling complex with CD14 and MD-2 is formed that activates various pro-inflammatory cytokines. The articles also summarize the role of viral non-structural protein, glycoproteins, and fusion proteins from Dengue, Ebola, SARS-CoV2, and RSV in the activation of TLR4 and present TLR4 as a potential therapeutic target to attenuate virus-induced cytokine storm.

Another review article by Woodson et al. summarized the new developments in Early Growth Response 1 (EGR1) biology. EGR1 is a transcription factor activated by different stimuli including viral infections. The review has highlighted the role of EGR1 during the infection of multiple DNA and RNA viruses. The viruses either activate or repress EGR1 which regulates the multiple signaling pathways of immune responses. The key genes that EGR1 regulates include host immune response genes like TNF, IL-6, IL-8, IL-2, MCP1, and multiple signaling pathways that influence virus replication and pathogenesis.

The research article by Du et al. has demonstrated the role of T cells in virus infection, particularly SAR-CoV2 infection. γδT cells don't require APC for antigen recognition and can either directly kill the infected cells by its secreted enzymes including granzyme or perforin and the use of TRAIL and Fas L induced apoptosis. The activated T cells trigger the innate immune response to release cytokines and restrict virus replication. Investigators in this research article have used a γδ-TCR model which revealed that activated γδT cells recognize SARS-CoV2 NSP8 peptides, highlighting the role of NSP8 in γδT cell-mediated immunity. The study provides an alternative approach to exploiting non-structural proteins to promote immune protection and reduce virus replication.

The review article by Wang, Li, et al. has summarized the advancements in the field of HIV and innate immune response. The minireview has highlighted the mechanisms by which HIV antagonizes and evades innate immune responses triggered during HIV infection. Of note, the review has summarized the role of the HIV capsid core in escaping immune surveillance. The capsid protein attenuates antiviral response through masking DNA from sensors like cGAS and through interaction with CPSF6, cyclophilin A, and nuclear pore complex proteins. The capsid protein also disables the antiviral activity of Trim5α.

The research article by Zhao et al. highlighted the role of Toll-like receptor 2 (TLR2) in Japanese encephalitis virus (JEV) induced inflammation and neuronal damage. In the study, a proteomic screen was performed which identified TLR2 as an important host protein responsible for activating JEV-induced inflammation. The authors further show that TLR2 acts through PI3K-AKT pathways in microglial cells. Overall, the study provides a novel signaling axis (TLR-PI3K-AKT) as a potential target to reduce JEV-induced neuro-inflammation and brain damage.

The research article by Zoellner et al. presented a study where they developed a synthetic cytokine/receptor system that phenocopies the natural cytokine signaling. Authors have shown that exchanged extracellular domains of IFNAR1/IFNAR2 with nanobodies that bind GFP and mCherry induce STAT1/2 signal transduction pathway. The study provides a novel platform to study the IFNAR signaling pathways and the role of tyrosine residues to activate downstream STAT1/2 phosphorylation. As a proof-of-concept, the authors show that the activation of synthetic receptors with synthetic ligands inhibits Vesicular Stomatitis Virus (VSV) replication. Therefore, such a synthetic cytokine receptor technology can be useful to study the virus-induced signaling pathways and to understand molecular events during virus-host interactions.

Another research article by Wang, Wan, et al. demonstrated that IFIT5 plays an important role in avian reoviruses (ARV) infection. The study showed that most upregulated ISGs in multiple tissues of chicken infected with ARV include IFIT5 and Mx. Over-expression of IFIT5 limits virus infection in chickens. Therefore, the study suggests the possible use of IFIT5 for targeting ARV infection and preventing ARV disease in chickens.

In conclusion, the published articles on this Research Topic have advanced our understanding of different aspects of the virus-host immune response. The collection of articles has summarized the latest developments in the field and highlighted the role of host and viral proteins as targets for therapeutic intervention. Such interventions can lessen the impact of virus-induced tissue damage and inflammatory responses and also in limiting virus-induced pathologies.

## Author contributions

All authors listed have made a substantial, direct, and intellectual contribution to the work and approved it for publication.
